# Data-Driven Engineering of Social Dynamics: Pattern Matching and Profit Maximization

**DOI:** 10.1371/journal.pone.0146490

**Published:** 2016-01-15

**Authors:** Huan-Kai Peng, Hao-Chih Lee, Jia-Yu Pan, Radu Marculescu

**Affiliations:** 1 Department of Electrical and Computer Engineering, Carnegie Mellon University, Pittsburgh, Pennsylvania, United States of America; 2 Department of Biomedical Engineering, Carnegie Mellon University, Pittsburgh, Pennsylvania, United States of America; 3 Google Inc., Mountain View, California, United States of America; Universitat Rovira i Virgili, SPAIN

## Abstract

In this paper, we define a new problem related to social media, namely, the data-driven *engineering* of social dynamics. More precisely, given a set of observations from the past, we aim at finding the best short-term intervention that can lead to predefined long-term outcomes. Toward this end, we propose a general formulation that covers two useful engineering tasks as special cases, namely, *pattern matching* and *profit maximization*. By incorporating a deep learning model, we derive a solution using convex relaxation and quadratic-programming transformation. Moreover, we propose a data-driven evaluation method in place of the expensive field experiments. Using a Twitter dataset, we demonstrate the effectiveness of our dynamics engineering approach for both pattern matching and profit maximization, and study the multifaceted interplay among several important factors of dynamics engineering, such as solution validity, pattern-matching accuracy, and intervention cost. Finally, the method we propose is general enough to work with multi-dimensional time series, so it can potentially be used in many other applications.

## 1 Introduction

The two distinct goals in scientific inquiry are understanding and engineering. Understanding is often associated with system analysis, modeling, and prediction, whereas engineering is often associated with design, control, and optimization. While understanding is fundamentally important, engineering often acts as the trigger for technological revolution. For example, although Kirchhoff discovered the basic circuit laws in 1845, VLSI revolution came only when the CMOS technology and its tool chain were invented more than a century later.

In the context of social networks, or more specifically, *social dynamics*, one would wonder what a similar triggering technology would be. Research in social dynamics has examined pattern discovery [1–4], modeling [5–7], and prediction [8–13]. Most, if not all, of this work, however, leans toward the understanding side. In contrast, the engineering side, which is more about control and targeted intervention of social dynamics, is far less explored. Even so, we argue that many engineering questions, if answered, have the potential to revolutionize the area of social networks. To illustrate, let us consider the following two engineering questions for social dynamics:

- *Pattern matching*: How can we manipulate social dynamics to follow a certain (successful) pattern in the future?
- *Profit maximization*: How can we maximize the long-term popularity of a hashtag using low-cost targeted intervention in the short-term?

The answers to such questions can impact significantly many aspects of social-media applications, e.g., marketing [14], politic [15], social mobilization [16], and disaster management.

Consequently, in this paper, we focus precisely on data-driven engineering approaches of social dynamics, where the main challenges are three-fold:

1. Define a problem formulation that is general enough to cover a *range of* engineering tasks.
2. Design a solution that is both reliable and efficient.
3. Provide a method to evaluate the quality of our solution using historical data, instead of using expensive and time-consuming *field experiments* [17].

To the best of our knowledge, this is the first work that offers the following contributions:

1. *Dynamics-engineering framework*: We propose a framework for data-driven dynamics engineering that consists of three components. First, we propose a general formulation, which includes both pattern matching and profit maximization as special cases. Second, using a deep-learning model, we derive a solution based on convex relaxation and quadratic-programming transformation, which is efficient and is guaranteed to converge to the global optimum. Finally, we propose a data-driven evaluation method instead of time-consuming and expensive field experiments [17].
2. *Experimental studies of pattern matching vs. profit maximization*: We experiment on both engineering tasks, i.e., pattern matching and profit maximization, using a Twitter dataset. For each task, we report and analyze the interesting tradeoffs that are critical to real-world dynamics engineering applications, including solution validity, pattern mismatch, intervention cost, and outcome popularity. Similarities and differences among the two tasks, together with their implications, are also discussed.

Finally, although in the present work we mainly focus on social dynamics, our formulation is general and can be applied to multi-dimensional time series. Consequently, the data-driven engineering methods we propose in this work can be, in principle, applied to other applications involving multi-dimensional time series, such as mobile context-aware computing, macroeconomics, or personalized medicine.

## 2 Related Work

Many authors have studied temporal patterns of social activities. These studies often cover various types of social dynamics, including the numbers of propagators and commentators [18], the breadth and depth of the propagation tree [19], the persistence of hashtags [15, 20], and general graph statistics (e.g., the graph diameter) [21–23]. Since our formulation is based on multi-dimensional time series, all of the above social dynamics can, in principle, apply our method for their specific engineering applications.

Another line of research targets the systematic pattern discovery of social dynamics. Much of this work conducts pattern mining using distance-based clustering. For example, the authors of [2] use spectral clustering for one-dimensional dynamics. Also, an efficient mean-shift clustering algorithm is proposed in [4] for multi-dimensional social dynamics. Other researchers use model-based methods to identify dynamics patterns. For example, the authors of [3] use a Gaussian Mixture model to analyze the proportions of readership before, at, and after the peak. Also, a deep-learning method that is capable of mining patterns of multiple time scales is proposed in [24]. In the present paper, we complement these previous works by actually *using* theses discovered patterns to engineer future dynamics.

Finally, many previous works are devoted to the modeling of social dynamics. Some of them are generative in nature [5–7] and define a probability distribution of social dynamics. There are also predictive models [8–13], where a probability distribution can be indirectly defined, e.g., by introducing Gaussian noise. Since our proposed formulation includes a probabilistic model as an independent component, in principle, all these models can be potentially plugged-in to our framework. We note that, however, it is generally difficult to use a predictive model alone to solve engineering tasks; this is because, by definition, intervention is not considered in dynamics prediction, but is required in dynamics engineering.

## 3 Method

First, we present our problem definition and formulation. We then incorporate the *Recursive Convolutional Bayesian Model* (*RCBM*) [24] into this formulation and derive a solution using convex relaxation and quadratic-programming (QP) transformation. Finally, we propose a novel data-driven evaluation method.

### 3.1 Problem Definition

**Social Dynamics**: In this work, we represent social dynamics as a *D*-dimensional time series $X\in R^{D\times T_{x}}$ that can characterize the propagation of any *information token*. As a running example, the dissemination of a Twitter hashtag can be characterized by the evolution of its three types (*D*=3) of users [4]: *initiators* who bring in information from the outside world, *propagators* who forward the information as it is, and *commentators* who not only forward the information, but also provide their own comments about it. All notations used in this paper are summarized using Table 1.

**Table 1:** Summary of notations used in this paper.

Variables	
*X*	observation dynamics; $X\in R^{D\times T_{x}}$.
*U*	intervention dynamics; $U\in R^{D\times T_{u}}$.
*V*	outcome dynamics; $V\in R^{D\times T_{v}}$.
*Y*	$Y=\left[U\;V\right]\in R^{D\times T_{y}}$.
*W*_*ik*_	the *k*-th filter matrix in the *i*-th layer; $W_{k}\in R^{D\times T_{w}}$.
*h*_*ik*_	the *k*-th activation vector in the *i*-th layer; $h_{ik}\in R_{+}^{T+T_{w}-1}$.
*D*	the dynamics dimensionality.
*T*_*x*_, *T*_*w*_, etc.	the temporal length of *X* or *W*, etc.
*σ*_*i*_, *β*_*i*_	parameters of *P*(*X*|*h*) and *P*(*h*), respectively.
*K*_*i*_	the number of filters in *i*-th level.
**x**,**y**, etc.	vectorization of *X* or *Y*, etc. (Eq 1).
**h**_*i*_	vector concatenation of ${\left\{h_{ik}\right\}}_{k=1}^{K_{i}}.$
*m*_*x*_, *m*_*y*_, etc.	length of **x** or **y**, etc.
*B*, *d*	parameters of the score function (Eq 2).
*C*_*cost*_, *C*_*reward*_	cost / reward parameters (Eqs 3 and 4)
*V*_*ref*_	pattern to be matched (Eq 3)
*ρ*	tradeoff parameter (Eqs 3 and 4).
*c*	max-pooling parameter.
*S*,**s**	max-pooling dummy variables; **s** = vec(*S*).
*Q*, *p*,*A*	canonical variables of Quadratic Programming
Special matrices and operations	
*I*_*n*_	*n*-by-*n* identity matrix
**1**_*m*×*n*_	*n*-by-*m* matrix with 1 in its elements.
**0**_*m*×*n*_	*n*-by-*m* matrix with 0 in its elements.
	Subscripts might be omitted for simplicity.
⊗	Specialized convolution in Eq 7
⊙	Kronecker product
vec(⋅)	vectorization of a matrix

**Dynamics-Engineering Problem (Informal)**: Given the *observation dynamics* $X\in R^{D\times T_{x}}$, find the best *intervention dynamics* $U\in R^{D\times T_{u}}$ such that some desired property of the *outcome dynamics* $V\in R^{D\times T_{v}}$ is optimized.

**Fig 1:**
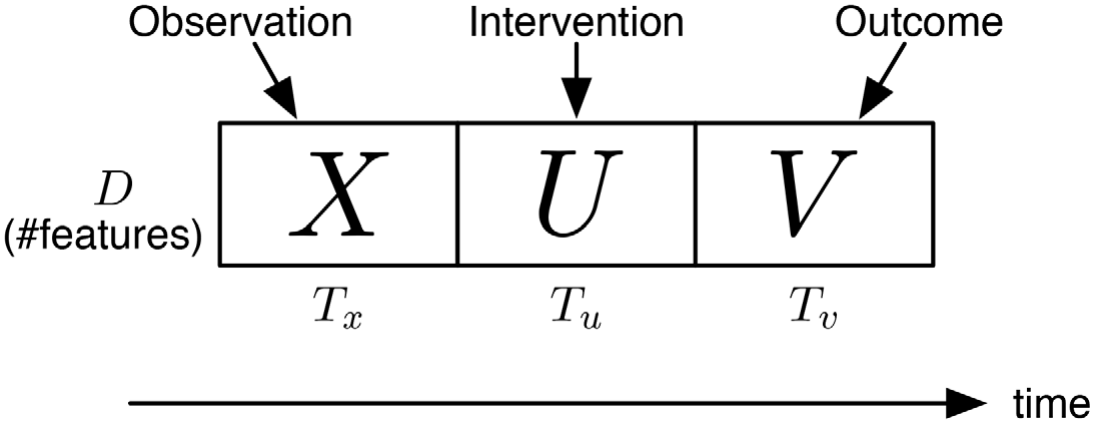
Illustration of the dynamics engineering problem. *X*: observation dynamics; *U*: intervention dynamics; *V*: outcome dynamics.

The problem is illustrated in Fig 1. In the figure, we split the dynamics (that correspond to, e.g., a hashtag) into three parts, *X*,*U*, and *V*, where the current time is at the end of *X* (i.e., immediately before *U*). The input of the dynamics engineering problem is *X*, plus the knowledge of the historical dynamics behavior (e.g., a model). The output of the problem is the best (recommended) intervention dynamics U*, such some properties of *V* is optimized. Ideally, we also want to obtain the projected outcome dynamics V*, i.e., as a result of U*.

Let us revisit our Twitter example mentioned above. Under this context, an example engineering problem is as follows: Given the observed numbers of the initiators, propagators, and commentators of a particular hashtag within the past 30 minutes (i.e., *X* in Fig 1), find the best possible intervention dynamics over the next 30 minutes (i.e., *U* in Fig 1), e.g., using incentive programs or direct promotions, such that the total readership of the hashtag is maximized in the following hours (i.e., *V* in Fig 1). Note that in the above problem definition, we assume that only the observation (*X*) is given, while both the ideal intervention (U*) and the projected outcome (V*) are to be identified.

Of note, the goal of this work is to find what the best intervention dynamics U* would *look like* given the input data. In other words, how to actually *implement* a particular intervention (e.g., using incentive programs, etc.) is a separate problem that is not covered in the present work.

**Score function**: To define the dynamics engineering problem formally, we first let *Y* = [*UV*]∈*R*^*D*×(*T*_*u*_+*T*_*v*_)^ denote the concatenation of the two matrices *U* and *V*. For example, if we have $U\;=\;\left[\begin{array}{cc} 1 & 2\\ 3 & 4 \end{array}\right]$ and $V\;=\;\left[\begin{array}{cc} 5 & 6\\ 7 & 8 \end{array}\right]$, then $Y\;=\;\left[\begin{array}{cccc} 1 & 2 & 5 & 6\\ 3 & 4 & 7 & 8 \end{array}\right]$. Note that this concatenation is merely for mathematical convenience: *U* and *V* still differ in their meanings and in the kinds of properties we want their corresponding solutions (U* and V*) to satisfy.

Moreover, let **y** = vec(*Y*) denote its vectorization (i.e., its transformation into a column vector):

$$y=\text{vec}\left(Y\right)=\text{vec}\left( [\begin{array}{c} -y_{1}^{T}-\\ \vdots \\ -y_{D}^{T}- \end{array}]\right)= [\begin{array}{c} y_{1}\\ \vdots \\ y_{D} \end{array}].$$ (1)

Using the same example above, we have $vec\left(\;\left[\begin{array}{cccc} 1 & 2 & 5 & 6\\ 3 & 4 & 7 & 8 \end{array}\right]\right)\;=\;{\left[1\;2\;3\;4\;5\;6\;7\;8\right]}^{T}$. Accordingly, we can reformulate the engineering problem as maximizing a *score function* defined as:

$$\text{score}\left(y\right)=y^{T}By+d^{T}y.$$ (2)

where *B* and *d* define the quadratic and linear parts of the score function, respectively. We note that this quadratic score function is general, in the sense that different goals can be achieved using various special cases. In particular, two interesting special cases are:

- *Pattern matching*: To achieve an ideal outcome *V*_*ref*_ while minimizing the cost associated with the required intervention *U*, one can maximize the following score function:
  $$\begin{array}{ccc} {\text{score}}_{\text{match}}\left(Y\right) & = & -\left(1-\rho \right)||V-V_{ref}{\left|\right|}_{f}^{2}-\rho \left\langle C_{cost},U\right\rangle \\ & = & y^{T}By+d^{T}y. \end{array}$$ (3)
  The first term denotes *mismatch* and will force *V* to match *V*_*ref*_; the second term denotes *cost* and will typically force values in *U* to be small. Here *ρ* ∈ [0, 1] controls the relative importance of mismatch versus cost. Moreover, *C*_*cost*_ encodes the relative expense of controlling different features at different time, whereas 〈*U*,*C*〉 = ∑_*ij*_ *U*_*ij*_ *C*_*ij*_ denotes the dot product between *U* and *C*. For example, suppose $C_{cost}\;=\;\left[\begin{array}{cc} 1 & 1\\ 2 & 2 \end{array}\right]$ and $U\;=\;\left[\begin{array}{cc} 1 & 2\\ 3 & 4 \end{array}\right]$, then 〈*C*_*cost*_,*U*〉 = 1×1 + 1 × 2 + 2 × 3 + 2 × 4 = 17. Returning to our Twitter example above, suppose that the first row of $U\;=\;\left[\begin{array}{cc} 1 & 2\\ 3 & 4 \end{array}\right]$ represents the numbers of propagators (i.e., one propagator at *t* = 1 and two propagators at *t* = 2) and that the second row represents the numbers of commentators (i.e., three commentators at *t* = 1 and four at *t* = 2), then assigning $C_{cost}\;=\;\left[\begin{array}{cc} 1 & 1\\ 2 & 2 \end{array}\right]$ is equivalent to specifying that it is twice as expensive to grow the number of commentators (Twitter users who spend time to leave comments) than to control the number of propagators (who simply click “retweet”), regardless of time. Finally, we note that Eq 3 is a special case of Eq 2. To check this, we can rewrite the second line of Eq 3 using $B=\left(1-\rho \right){\hat{I}}_{v}^{T}{\hat{I}}_{v}$, $d=\text{vec}(\left[-\rho C_{u}\;2\left(1-\rho \right)V_{\text{ref}}\right])$, and ${\hat{I}}_{v}=I_{D}⊙\left(\left[0_{T_{v}\times T_{u}}\;I_{T_{v}}\right]\right)$. Here ⊙ denotes the Kronecker product (see Table 1 for a summary of notations).
- *Profit maximization*: To maximize the reward associated with the outcome *V* while minimizing the cost associated with the intervention *U*, one can maximize the following score function:
  $$\begin{array}{ccc} {\text{score}}_{\text{profit}}\left(Y\right) & = & -\rho \left\langle C_{cost},U\right\rangle +\left(1-\rho \right)\left\langle C_{reward},V\right\rangle \\ & = & d^{T}y, \end{array}$$ (4)
  The first term denotes *cost* and will typically force the values in *U* to be small; the second term denotes *reward* and will typically force the values in *V* to be large. Similarly to the above task, we use *C*_*cost*_ to encode the relative cost and use *C*_*reward*_ to encode the relative reward of different dimensions and time. Following the above Twitter example, assigning $C_{reward}\;=\;\left[\begin{array}{cc} 1 & 3\\ 1 & 3 \end{array}\right]$ is equivalent to specifying that it is three times more rewarding to acquire a user (either a propagator or a commentator) at *t* = 2 compared to acquiring a user at *t* = 1, regardless of the type of the user. Like the case of Eq 3, *ρ* controls the relative importance of cost versus reward. We note that Eq 4 is another special case of Eq 2. To check this, we can rewrite the second line of Eq 4 using $d=\text{vec}(\left[-\rho C_{cost}\;\;\left(1-\rho \right)C_{reward}\right])$.

**Formal Definition**: While maximizing the score function, we make two implicit assumptions: (1) there exists a temporal dependency among *X* and *Y* = [*U V*], and (2) the solution we come up with needs to follow that dependency. Accordingly, we propose the following formal definition of our problem:

**Dynamics-Engineering Problem (Formal)**: Given observation *X*, a probabilistic model *P*(⋅), and a score function score(Y), find:

$$Y^{*}=\left[U^{*}\;V^{*}\right]=\underset{Y}{arg max}\;\;\log P\left(Y|X\right)+\lambda \text{score}\left(Y\right).$$ (5)

Here *P*(⋅) denotes the log-likelihood using a probabilistic model that captures the temporal dependencies of the social dynamics. In other words, while the second term (i.e., the score function) takes care of the specific engineering task, the first term (i.e., log *P*(*Y*|*X*)) makes sure that the solution still conforms with the temporal dependency of the social dynamics. Moreover, λ ≥ 0 is a balancing parameter that controls the relative importance of fitting the probability distribution *P*(⋅) versus maximizing the score. Of note, the selection of λ is crucial and will be described in detail later.

We note that our proposed problem definition is *general* yet *precise*. Indeed, it can incorporate any combination of *P*(⋅) and score(Y) functions, in which any different combination corresponds to a different engineering task. Also, once this combination is given, the engineering problem is mathematically precise.

### 3.2 Deep-Learning Model

In principle, any probabilistic model of social dynamics can be plugged into the likelihood term *P*(⋅) in Eq 5. In this work, we use the *Recursive Convolutional Bayesian Model (RCBM)* that we proposed recently [24]. As it will be shown in the experimental section, the choice of this model makes a big difference.

According to RCBM, the basic generation process for dynamics *X* is:

$$\begin{array}{ccc} P(h;\beta ) & = & \frac{1}{\beta }\exp\left(\frac{\sum_{k}\left|\right|h_{k}{\left|\right|}_{1}}{-\beta }\right)\\ P(X|h;W,\sigma ) & = & \frac{\sqrt{2}}{\sigma \sqrt{\pi }}\exp\left(\frac{||X-\sum_{k}W_{k}⊗h_{k}{\left|\right|}_{F}^{2}}{-2\sigma^{2}}\right), \end{array}$$ (6)

More specifically, RCBM assumes that dynamics *X* (or more generally, the concatenation of [*X U V*]) are generated from making “scaled copies” of the *filter matrices* *W*_*k*_’s, where the time shift and the scaling of these copies are determined by the sparse *activation vectors* *h*_*k*_’s. Such a “scale-and-copy” operation is carried out using the operator ⊗ in Eq 6, which denotes a dimension-wise convolution defined as:

$$\left(W⊗h\right)\left[d,t\right]=\sum_{s=1}^{T_{w}}h\left[t+T_{w}-s\right]\cdot W\left[d,s\right]\;\;\;\forall d,t.$$ (7)

We note that this operator differs from the conventional matrix convolution used in [25, 26]. Effectively, ⊗ does *D* 1-D convolutions between each row of *W* and the entire *h*, and puts back the results to each row of the output matrix separately.

By stacking multiple levels of the basic form in Eq 6, we can construct a deep-learning architecture:

$$\begin{array}{ccc} P(X,h) & = & \prod_{l}P\left(X_{l}|h_{l};W_{l},\sigma_{l}\right)P\left(h_{l};\beta_{l}\right)\\ & = & \frac{1}{Z}\exp\left(\sum_{l}\frac{\left|\right|X_{l}-\sum_{k}W_{l,k}⊗h_{l,k}{\left|\right|}_{F}^{2}}{-2\sigma_{l}^{2}}+\frac{\sum_{k}\left|\right|h_{l,k}{\left|\right|}_{1}}{-\beta_{l}}\right). \end{array}$$ (8)

The key of this construction is building the upper-level dynamics *X*_*l*_ by *max-pooling* [25, 26] the lower-level activation vectors *h*_*l*−1,*k*_. This essentially takes the maximum value over *c* consecutive values of the lower-level activation vectors. This operation introduces non-linearity into the model, which is key for the superior performance of deep-learning methods [24–26].

In [24], we have derived an efficient algorithm for learning RCBM and have demonstrated several applications (i.e., pattern discovery, anomaly detection) in *understanding* social dynamics. In the present work, we shift focus from understanding to *engineering* social dynamics.

### 3.3 RCBM-based Formulation

By writing down the conditional probability *P*(*Y*|*X*) using the joint probability specified in Eq 8 and then plugging *P*(*Y*|*X*) into the first term of Eq 5, the optimization problem in Eq 5 can be explicitly written as:

$$\begin{array}{cc} \underset{Y,\;h_{1},\;h_{2},\;S}{arg min} & \frac{1}{2}||\left[X\;Y\right]-\sum_{k}W_{1k}⊗h_{1k}{\left|\right|}_{F}^{2}+\frac{\sigma_{1}^{2}}{\beta_{1}}\sum_{k=1}^{K_{1}}\left|\right|h_{1k}{\left|\right|}_{1}\\ & +\frac{1}{2}||MP\left(h_{1}\right)-\sum_{k}W_{2k}⊗h_{2k}{\left|\right|}_{F}^{2}\\ & +\frac{\sigma_{2}^{2}}{\beta_{2}}\sum_{k=1}^{K_{2}}\left|\right|h_{2k}{\left|\right|}_{1}-\lambda \left(y^{T}By-d^{T}y\right)\\ \text{s}.\text{t}. & h_{1k}\geq 0,\;h_{2k}\geq 0andy\geq 0. \end{array}$$ (9)

Here, a two-level RCBM is presented for illustration purposes, though the optimization formulation for a multilevel RCBM can be similarly derived. The max-pooling operation MP(⋅) is defined as

$$MP\left(h_{1}\right)\left[k,t\right]=\max_{i\in 1,\;\cdots ,\;c}h_{1k}\left[\left(t-1\right)c+i\right],$$ (10)

where **h**_1_ is the vector concatenation of ${\left\{h_{ik}\right\}}_{k=1}^{K_{i}}$. As mentioned in Section 3.2, MP(⋅) is the key that enables RCBM (or more generally, any convolutional deep-learning method) to learn the nonlinear features of the series. However, it also imposes significant difficulties in optimization by making the problem non-differentiable and non-convex. Consequently, the problem in Eq 9 is not only difficult to solve, but also prone to getting stuck at suboptimal solutions.

### 3.4 Convex Relaxation and QP transformation

To solve the difficulty resulted from max-pooling, we propose the following convex relaxation:

$$\begin{array}{cc} \underset{Y,\;h_{1k},\;h_{2k},\;S}{arg min} & \frac{1}{2}||\left[X\;Y\right]-\sum_{k}W_{1k}⊗h_{1k}{\left|\right|}_{F}^{2}+\frac{\sigma_{1}^{2}}{\beta_{1}}\sum_{k=1}^{K_{1}}\left|\right|h_{1k}{\left|\right|}_{1}\\ & +\frac{1}{2}||S-\sum_{k}W_{2k}⊗h_{2k}{\left|\right|}_{F}^{2}+\frac{\sigma_{2}^{2}}{\beta_{2}}\sum_{k=1}^{K_{2}}\left|\right|h_{2k}{\left|\right|}_{1}\\ & -\lambda (y^{T}By+d^{T}y)\\ \text{s}.\text{t}. & h_{1k}\geq 0,\;h_{2k}\geq 0andY\geq 0,\\ & h_{1k}\left[\left(t-1\right)c+i\right]\leq S\left[k,t\right]\\ & S\left[k,t\right]\leq \sum_{i=1}^{c}h_{1k}\left[\left(t-1\right)c+i\right]. \end{array}$$ (11)

The idea behind this relaxation consists of introducing a new variable *S* as the surrogate of MP(⋅). Furthermore, we substitute the *equality* constraints specified in Eq 10 with two sets of *inequality* constraints, i.e.,

$$\max_{i\in 1,\;\ldots ,\;c}h_{1k}\left[\left(t-1\right)c+i\right]\leq S\left[k,t\right]\leq \sum_{i=1}^{c}h_{1k}\left[\left(t-1\right)c+i\right].$$

In other words, instead of forcing *S* to equate $S\left[k,t\right]=\max_{i\in 1,\;\ldots ,\;c}h_{1k}\left[\left(t-1\right)c+i\right]$, i.e., the maximal value among the consecutive *c* values, we now constrain it to be larger than or equal to the maximal value, but smaller than or equal to the sum of those *c* values.

We note that the problem in Eq 11 is now *jointly* convex in *Y*, *h*_1_, *h*_2_ and *S*, since the objective function is convex and all constraints are linear. Moreover, since the objective is differentiable, a possible approach to solve Eq 11 is using the proximal method [27]. It turns out, however, the projection functions corresponding to the constraints in Eq 11, which are required in the proximal method, are difficult to derive.

We solve this issue by noting that the objective function of Eq 11 is quadratic with only linear constraints. Therefore, in principle, there exists a quadratic programming (QP) transformation that is equivalent to Eq 11. The explicit form and the mathematical details of this QP transformation is described in S1 Appendix. We note that, since the problem is jointly convex, QP is guaranteed to find an approximate solution in polynomial time. In our experiments, the QP has around 1000 variables and the problem gets solved in just a few seconds.

### 3.5 Data-driven evaluation

For many methods in modeling and prediction, cross-validation [28] is the standard way for evaluating solutions and selecting parameters. However, cross-validation cannot be directly applied to our dynamics engineering problem, because the properties of a “good solution” for modeling and prediction is well-known. For example, a good modeling solution will have high data likelihood and a good prediction solution will be highly accurate. For our dynamics-engineering problem, however, such a property is less obvious.

For the dynamics engineering problem, we argue that the key property of a good solution consists of *combining* a high score and a high *validity*, where the latter can be roughly defined as how well the solution is supported by historical samples that achieve high scores. To show that having a high score alone is not sufficient, consider the case when λ → ∞ in Eq 5. In this case, the optimization will produce the highest possible score, while completely ignoring the likelihood term in Eq 5. As a result, the optimization will produce a solution that does not possess any inherent temporal dependency of the data. In this case, the projected outcome V* would be unlikely to happen in the real world even if the suggested intervention U* is implemented.

#### 3.5.1 Validity

As mentioned above, the informal definition of validity is how well the solution is supported by historical samples that achieve high scores. To formally define *validity* γ, two important components are: (1)$\hat{P}$ that denotes the density function capturing what the high-scoring dynamics look like in historical data, and (2) *q*_0_ that denotes a carefully chosen threshold. More precisely, $\hat{P}\left(\cdot \right)$ and *q*_0_ are constructed in four steps:

1. Evaluate the value of the score function using all historical samples ${\left\{\left[X_{i},Y_{i}\right]\right\}}_{i=1}^{N}$, rank them according to their evaluated values, and then keep only the *N*_0_ top-scoring samples.
2. Use the first half ${\left\{\left[X_{i},Y_{i}\right]\right\}}_{i=1}^{\left\lfloor \frac{N_{0}}{2}\right\rfloor }$ to construct a kernel density estimator [29]:
  $$\hat{P}\left(X,Y;h\right)\propto \sum_{i}\exp\frac{||\left[X,Y\right]-\left[X_{i},Y_{i}\right]{\left|\right|}^{2}}{-2\omega^{2}}.$$
3. Use the second half ${\left\{\left[X_{i},Y_{i}\right]\right\}}_{i=\lfloor \frac{N_{0}}{2}\rfloor +1}^{N_{0}}$ to choose the value of *ω* that has the highest data likelihood.
4. Use the second half to calculate *q*_0_, such that only a small fraction (e.g., 5%) of samples among the second half has $\hat{P}\left(X,Y;h\right)<q_{0}$.

With $\hat{P}\left(\cdot \right)$ and *q*_0_ defined, we can define the *validity* γ corresponding to a solution Y*(λ) (i.e., solution of Eq 5 using a specific value of λ) as:

$$\gamma \left(\lambda \right)=\log\frac{\hat{P}\left(\left[X,Y^{*}\left(\lambda \right)\right]\right)}{q_{0}}.$$ (12)

Then we can use γ as a convenient measure, such that γ ≥ 0 indicates that, according to historical high-scoring data, the solution is “realistic enough”.

The main idea behind the above procedure is to construct $\hat{P}\left(X,Y\right)$ as the density estimator of the high-scoring historical samples, and then construct *q*_0_ as the *quantile estimator* (e.g., at 5%) for the empirical distribution of {*P*_*i*_}_*i*_. Here $P_{i}=\hat{P}\left(X_{i},Y_{i}\right)$ denotes the value of $\hat{P}\left(\cdot \right)$ evaluated using *X*_*i*_ and *Y*_*i*_. Therefore, when the density of a solution $\hat{P}\left(\left[X,Y^{*}\left(\lambda \right)\right]\right)$ is larger than *q*_0_ (i.e., when γ ≥ 0 in Eq 12), we call this solution as being “realistic enough”, because it is more likely (i.e., more realistic) than the 5% most-unlikely high-scoring historical samples.

We note that in the construction of γ, and in particular, $\hat{P}\left(\cdot \right)$ and *q*_0_, we do not use the entire training set. The underlying reason is that a realistically good solution can be very rare. In other words, it is by design that validity should measure how well a solution is supported by historical samples *that achieve high scores*, instead of historical samples in general. Consequently, in principle, *N*_0_ should be selected as a small fraction (e.g, 10%) of the size of the historical samples.

Finally, we note that $\hat{P}\left(X,Y\right)$ and *q*_0_ depend on the partitioning in the second and the third steps, which, according to Eq 12, can also affect γ(λ). A simple way to remove such a dependency is to use multiple random partitionings, obtain the corresponding copies of $\hat{P}\left(X,Y\right)$’s and *q*_0_’s, and then calculate the average value of Eq 12 using all these copies.

#### 3.5.2 Selection of λ

With validity defined, we are now ready to select λ. As mentioned before, it should be the combination of high validity and high score. A key observation from Eq 5 is that one can make the score larger by making λ larger. Therefore, while there may be many potential ways to do it, we propose the following method:

$$\begin{array}{cc} \underset{\lambda }{arg max} & \;\lambda \\ \text{s}.\text{t}. & \gamma (\lambda )\geq 0, \end{array}$$ (13)

where the idea is that conditioned on the solution being (sufficiently) valid, we want its score to be as high as possible. Finally, we use a Twitter dataset to demonstrate the interplay among λ, validity (γ), and score while engineering social dynamics in the next section.

## 4 Experimental Results

For experimental results, we first describe our dataset, the overall setup, and two baseline methods. Then, we present experimental results on two engineering tasks: pattern matching and profit maximization.

### 4.1 Dataset

We use the Twitter dataset from [2] that consists of 181M postings during June to December of 2009 from 40.1M users and 1.4B following relationships. With this dataset, hashtags are used to enumerate the information tokens that carry social dynamics. We filter out “low-traffic” hashtags by selecting only the ones with at least 100 total usages around the 90 minutes during their peak times, yielding a 10K-sample dataset of social dynamics. We then sort these samples according to their peak time. The first 9K samples are used as the training set, i.e., for model training and the construction of $\hat{P}\left(\cdot \right)$ and *q*_0_ (mentioned in Section 3.6), whereas the remaining 1K samples are reserved for testing. This data partitioning scenario ensures that all training data occurs prior to testing data, i.e, no “future data” is used while testing. Finally, for all hashtag samples, we measure the dynamics in units of 3 minutes, where the first 30 minutes are the observation dynamics (*X*), the middle 30 minutes are the intervention dynamics (*U*), and the last 30 minutes are the outcome dynamics (*V*).

We characterize each social dynamic using its five types of users [4]. *Initiators* denote the users who use this keyword before any of his or her friends did. *First-time propagators* and *first-time commentators* denote the users who retweet and tweet, respectively, about this keyword after his or her friends using the same keyword before. *Recurring propagators* and *recurring commentators* denote the users who retweet and tweet, respectively, the same keyword that they used before. Of note, it means that *X*,*U*, *V* ∈ *R*^5×10^ because now each variable has five features and ten timesteps (i.e., three minutes per timestep).

### 4.2 Setup

We conduct experiments on two types of engineering tasks, namely, by solving Eq 5 using two distinct score functions: the one in Eq 3 for pattern matching and the one in Eq 4 for profit maximization. For pattern matching, we set *C*_*cost*_ = **1**_*D*×*T*_*u*__ in Eq 3 to assume a uniform intervention cost in time and for different types of users. Similarly, for profit maximization, we set *C*_*cost*_ = **1**_*D*×*T*_*u*__ and *C*_*reward*_ = **1**_*D*×*T*_*v*__ in Eq 4. Of note, the assignment of *V*_*ref*_ in Eq 3 depends on the particular experiment and will be detailed later.

In order to analyze the interplay and tradeoffs critical to real-world engineering applications, for each task, we conduct analyses along the following four directions:

1. Interplay between validity γ (Eq 12) and the optimization parameter λ (Eq 5).
2. Tradeoff of individual terms in the score functions. In particular, for pattern matching (Eq 3), it includes cost (<*C*_*cost*_,*U*>) and mismatch ($||V-V_{ref}{\left|\right|}_{f}^{2}$); for profit maximization (Eq 4), it includes cost (<*C*_*cost*_,*U*>) and reward (<*C*_*reward*_,*V*>).
3. Comparison between “real” vs. engineered cases. The motivation behind this analysis is to quantify the potential benefits as a result of purposeful engineering, compared to what happened in reality.
4. A case study.

### 4.3 Baseline Methods

**AR**: Our first baseline is to substitute the likelihood term in Eq 5 with another one using the Autoregressive model (AR). AR is commonly used in time-series forecasting and is defined as:

$$x_{t}=\sum_{i=1}^{p}\Phi_{i}x_{t-i}+\epsilon_{t}$$ (14)

Here *x*_*t*_ ∈ *R*^*D*×1^ denotes the multivariate features at time *t*; $\epsilon_{t}∼N\left(0,\;\Sigma \right)$ denotes the i.i.d. multivariate Gaussian noise; *Φ*_*i*_’s denote the matrices for modeling the dependency between the current dynamics and its history back to *p* steps, where we set *p* = 10. Details of solving Eq 5 with the first term using AR is given in S1 Appendix. While this baseline fits perfectly in our proposed framework, its restrictive linear generative model may limit its performance.

**NN**: Our second baseline is based on the nearest-neighbor (NN) search. The idea is to search within the training data for the top 5% samples that are the most similar to the given observation *X* (using Euclidean distance). Then the solution Y* is obtained using the {*U*, *V*} part of the highest-scoring sample within that subset. The advantage of NN is that, unlike other methods, it doesn’t have a concern about validity, i.e., whether the solution is realistic or not, because the solution is generated from real dynamics that happened in the past. However, its disadvantage is that not all historical dynamics matches the observation *X* and maximizes the score function at the same time. Consequently, the score of NN’s solution may be low or unstable.

### 4.4 Experiment 1: Pattern Matching

In our first experiment, i.e., pattern matching, we are given the observation *X*_*i*_ of every test sample and we aim at matching a single *V*_*ref*_. This *V*_*ref*_ is defined as the average outcome dynamics of the top 2% samples in the training set with the highest long-term popularity ||*V*||_1_. We conduct dynamics engineering using all test samples and analyze the resulting validity, cost, and mismatch.

#### 4.4.1 Validity (γ) vs. λ

**Fig 2:**
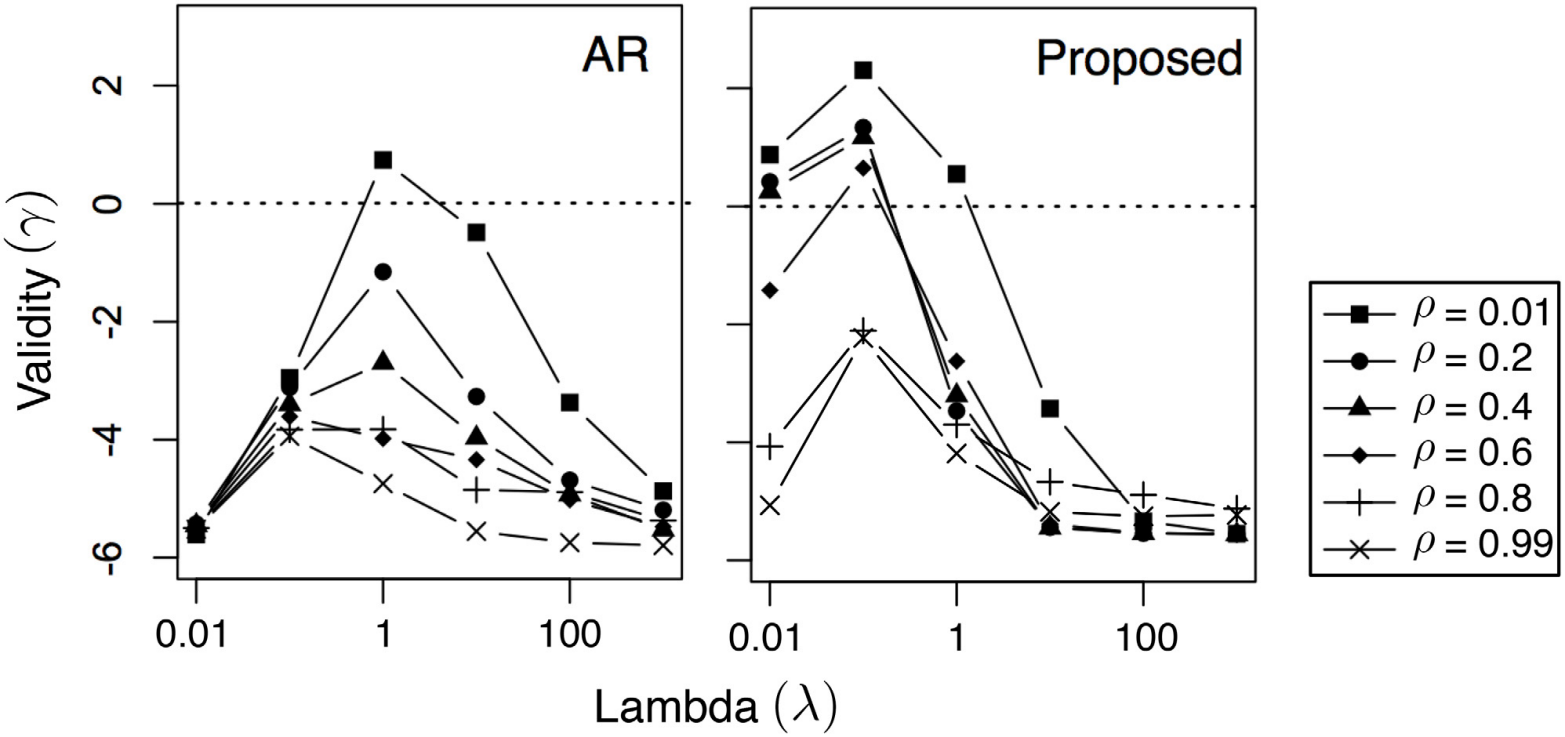
Influence of λ on solution validity γ using different values of *ρ*. The dotted lines mark λ ≥ 0, above which the solution is considered valid.

In Fig 2, we analyze the effect of different values of λ on the average solution validity γ. The dotted horizontal lines marks γ = 0, above which the solution is considered valid. Note that NN is not included here since it doesn’t require the selection of λ. From the figure, the observations are twofold. First, there is indeed a range of λ that produces valid solutions. In particular, that range changes with the value of *ρ*: the lower *ρ* is, the larger the range is. This is because a lower *ρ* puts more emphasis on minimizing mismatch instead of cost (see Eq 3). While there is nothing unrealistic about the pattern that needs to be matched, matching it using an extremely low cost (i.e., using a large *ρ*) can be unrealistic. Second, the proposed method outperforms the AR baseline in terms of validity, since the results using the proposed method have a lot more cases above the dotted line (indicating γ ≥ 0) compared to AR. This is because the proposed method incorporates RCBM that can effectively capture non-linear features, whereas AR is a linear model.

#### 4.4.2 Cost-mismatch Tradeoff

**Fig 3:**
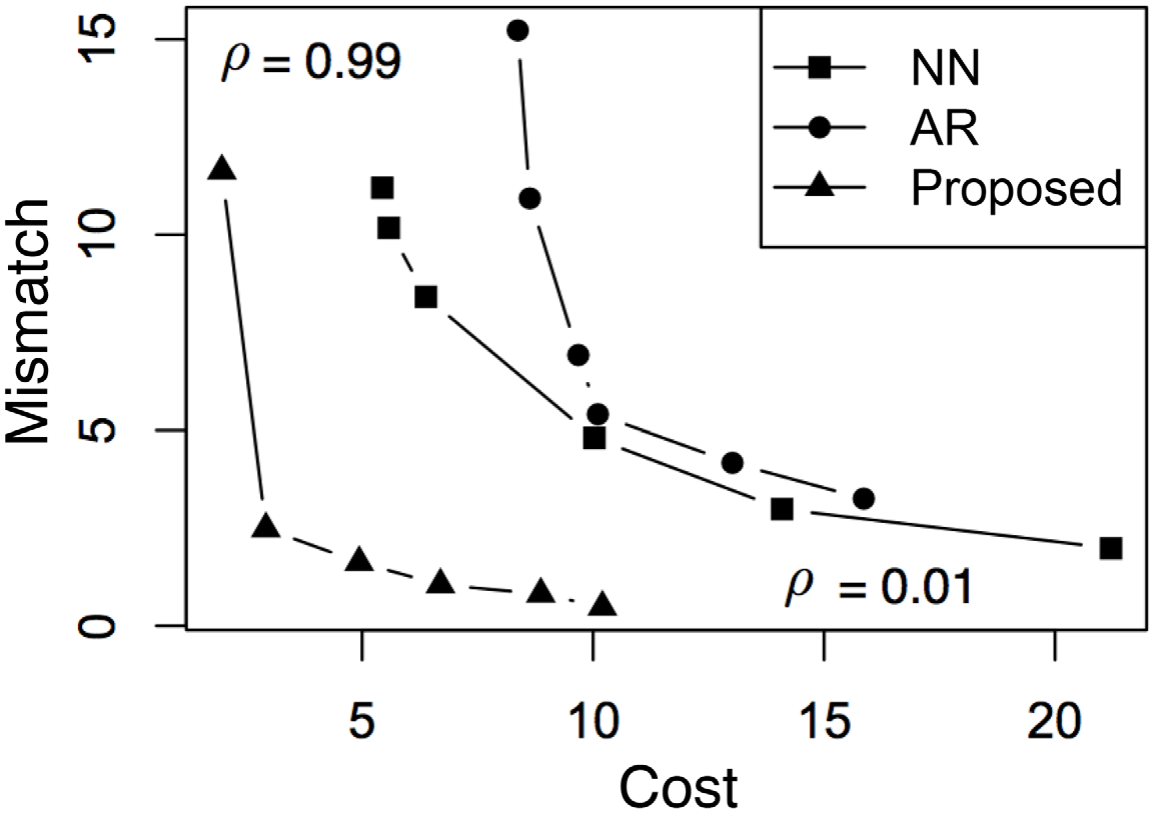
Tradeoff between cost and mismatch using different values of *ρ*. For each method, the point in the lower-right corner corresponds to the case of *ρ* = 0.01, whereas the point in the upper-left corresponds to the case of *ρ* = 0.99.

In Fig 3, we further analyze the tradeoff of using different values of *ρ* where λ is selected using Eq 13. In cases when there is no λ that satisfies γ(λ) ≥ 0, we select arg max_λ_ γ(λ) instead. The results of average cost versus mismatch using all three methods are summarized in Fig 3. For each method, the point in the lower-right corner corresponds to the case of *ρ* = 0.01, whereas the point in the upper-left corresponds to the case of *ρ* = 0.99. From the figure, the newly proposed method consistently makes the best tradeoff: with the same mismatch, it achieves a lower cost; with the same cost, it achieves the lower mismatch. The reason for this is twofolds: for AR, its linear model is too restrictive to reach either of the two objectives; for NN, the samples in the subset of training data that matches the given observation does not necessarily have a high score. Another interesting observation is that NN seems to make better tradeoffs compared to AR. This shows that the selection of a good generative model is crucial for dynamics engineering.

#### 4.4.3 Constrained Cost Minimization

In order to demonstrate the potential benefits of purposeful engineering, we use a slightly different setting. While for each test sample *i*, we are still given the observation part *X*_*i*_, we set *V*_*ref*_ = *V*_*i*_, i.e, its own outcome dynamics. This setting allows us to compare the performance of the matching algorithms, in terms of cost, with what actually happened in reality, assuming that each test sample was actually performing a (perfect) matching task without consciously considering minimizing the cost.

Since the *real* case achieves a “perfect match”, we need to constrain the engineering algorithms such that they can be compared on the same footing. Therefore, we enforce an additional constraint ||V*−*V*_*ref*_||_1_ ≤ *pDT*_*v*_ where *p* = 5%. In other words, after going through every test sample, each algorithm will have its own fraction of valid answers, and only the valid answers will be compared to the same set of samples, in terms of cost, to the real case. For AR and the proposed method, a valid answer must satisfy this constraint *on top of* satisfying γ ≥ 0.

**Fig 4:**
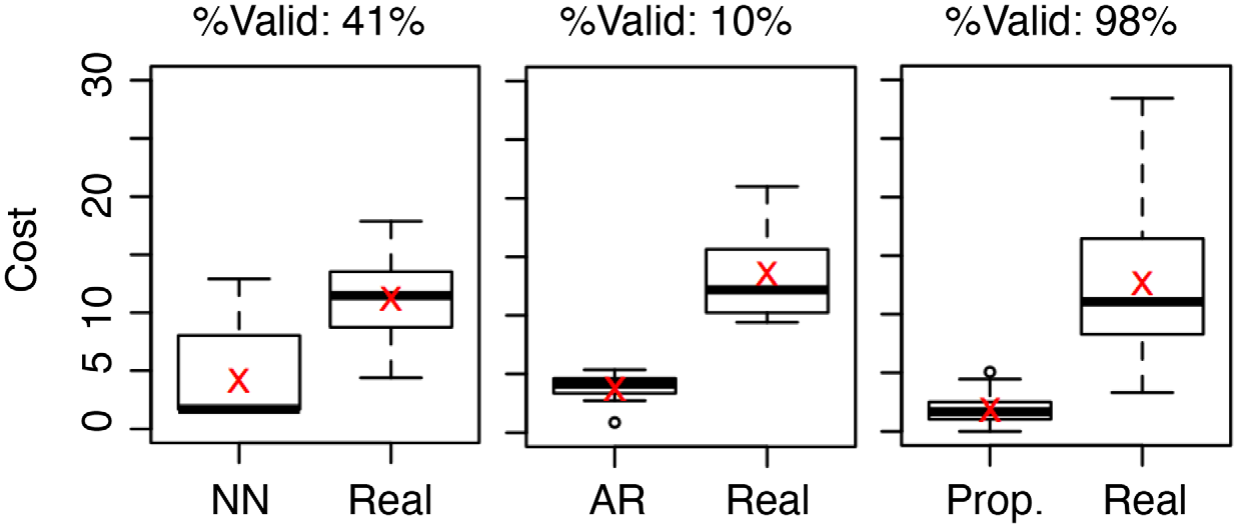
Cost distribution of valid answers using different methods. The cost distribution of each method is contrasted with that of the same set of samples in the real case. The mean values are marked using red crosses.

Fig 4 summarizes the results where the fraction of valid answers are annotated at the top and the mean values are marked using red crosses. From the figure, we note that NN produces valid answers for 41% of the test samples, whereas the number is 10% for AR and 98% for the proposed method. Also, the mean cost among the valid solutions using NN is 4.34, compared to 4.82 for AR and 2.31 for the proposed method. In other words, the proposed method achieves not only the largest fraction of valid solutions, but also the lowest average cost for that larger fraction. Note that the cost produced by NN has a high variation, confirming our expectation in Section 4.3. Finally, they all achieve lower cost than the corresponding samples in the real case, which is somewhat expected because the real cases were not consciously minimizing the cost. This further highlights the cost-saving potential of these dynamics-engineering methods.

#### 4.4.4 Case Study

**Fig 5:**
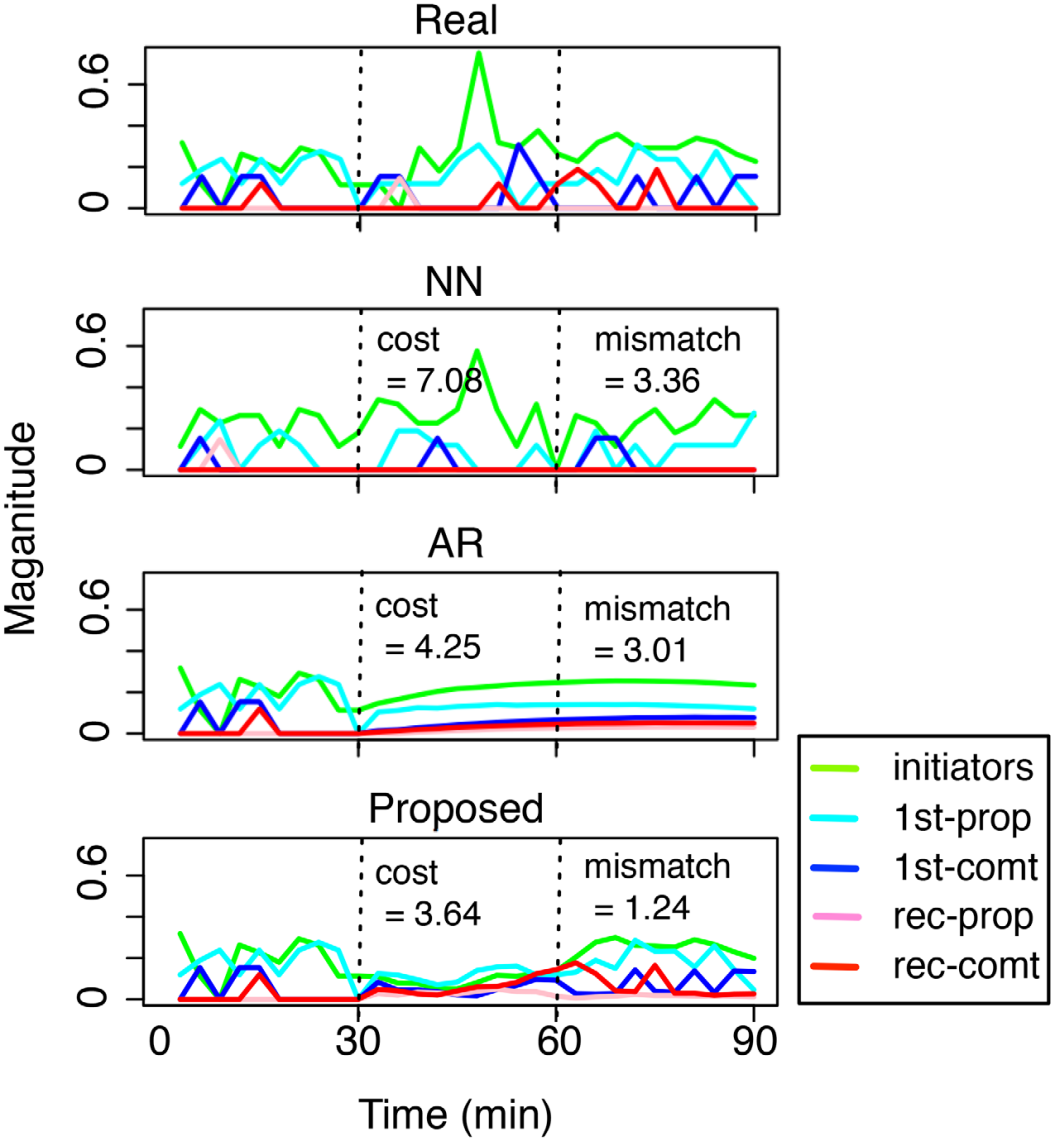
Case study: real versus the suggested dynamics using different methods. A good solution is characterized by low cost (during *t* ∈ [30, 60]) and low mismatch (during *t* ∈ [60, 90]). The X-axis denotes time (in minutes) and the Y-axis denotes the normalized number of different types of users.

To gain further insights, we pick a test case where all three methods produce valid solutions from the experiment of Fig 4 and plot their suggested solutions in Fig 5. For AR and the proposed method, since their solution only cover the last 60 minutes, their first 30 minutes are copied from the real case. From the real case, we see that it is a rather sustained dynamics that seems to be full of interactions among different types of users. To compare among different solutions (i.e., NN, AR, and Proposed), we note that the ideal pattern-matching should achieve both low cost during *t* ∈ [30, 60] and low mismatch during *t* ∈ [60, 90].

The solution produced by NN, although seems to match the real case in its general shape, it produces a moderate mismatch. Further, the cost of its suggested intervention is the highest among the three. AR, on the other hand, produces a very smooth dynamics that does not match the general shape of (the third part of) the real case, although the mismatch is quantitatively comparable to that of NN. Moreover, although its cost is relatively low, the dynamics doesn’t look real: in fact, its solution validity γ is 0.02, i.e., barely passes 0.

Finally, the proposed method produces a recommendation that best matches the third part of the real case, while also producing the lowest-cost intervention. A closer inspection shows that although the magnitude of the intervention dynamics (i.e., the second part) is generally low, it seems to consciously keep an interesting proportion and interaction among different types of users, i.e., initiators and first-time propagators around *t* = 50, 1st-time commentators around *t* = 55, and recurring commentators around *t* = 65. This shows that the key features for successful dynamics engineering are not necessarily unique and may involve the interaction of multiple features. This is made possible because the proposed recommendation explicitly use the patterns (i.e., the filters *W*’s in Eqs 9 and 8) at different temporal scales that are learnt directly from data. Consequently, the proposed method is able to recommend low-cost, good-matching solutions while still making the suggested dynamics follows the intrinsic temporal dependencies from the data.

### 4.5 Experiment 2: Profit Maximization

In our second experiment, i.e., profit maximization, we are given the observation *X*_*i*_ of every test sample and aim at maximizing the long-term popularity (reward) ||*V*||_1_ with minimum cost ||*U*||_1_. Again, we conduct dynamics engineering using all test samples and analyze the resulting validity, cost, and reward.

#### 4.5.1 Validity (γ) vs. λ

**Fig 6:**
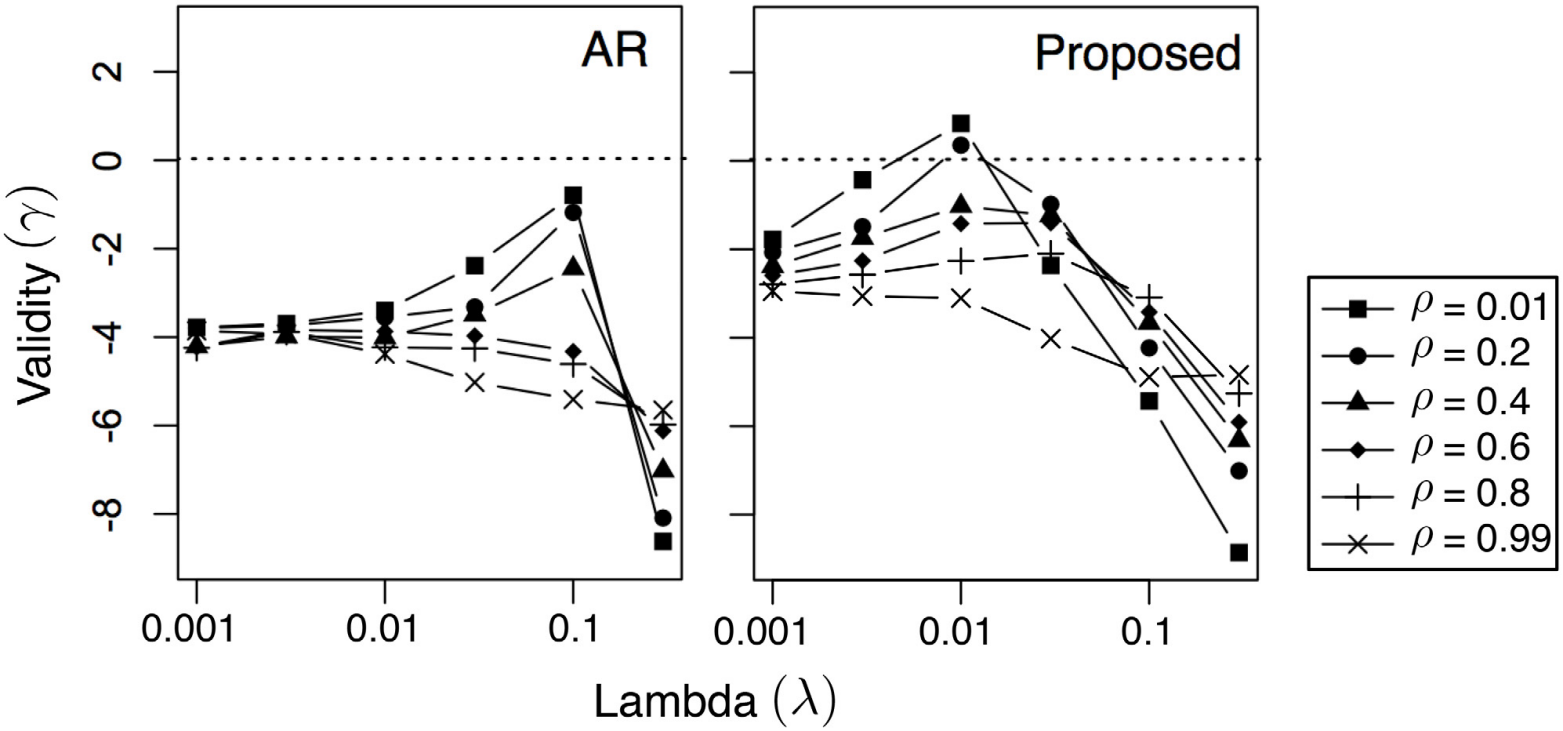
Influence of λ on solution validity γ using different values of *ρ*. The dotted lines mark λ ≥ 0, above which the solution is considered valid.

In Fig 6, we present the effects of different values of λ on the average solution validity γ, where the dotted horizontal lines marks γ = 0 (above which the solution is considered valid). There are two observations in Fig 6 that are consistent with Fig 2. First, there is a range of λ that produces valid solutions; the lower *ρ* is, the easier to produce valid solutions. Since a lower *ρ* puts more emphasis on reward instead of cost (see Eq 4), it suggests that the key to produce good solutions is putting a low (numerical) weight on cost. Second, the proposed method outperforms the AR baseline in terms of validity, indicating that the proposed method produces a lot more valid cases (γ ≥ 0) compared to AR. This confirms that the proposed method incorporates RCBM that can effectively capture non-linear features, whereas AR is a linear model.

Interestingly, there are also three observations in Fig 6 that are different from that of Fig 2. First, the validity value γ is generally smaller, indicating that as a task, profit maximization is more challenging than pattern matching. Second, the best λ’s that correspond to the highest γ’s are also about 10*X* smaller than that of Fig 2. It suggests that in profit maximization, one must put more emphasis on the likelihood instead of the score in Eq 5. Finally, the range of λ that is above zero (λ ≈ 0.01) is much more narrow than the case of Fig 2 (λ ∈ [0.01, 1]). This confirms that the task of profit maximization is more challenging than pattern matching.

#### 4.5.2 Reward-cost Tradeoff

**Fig 7:**
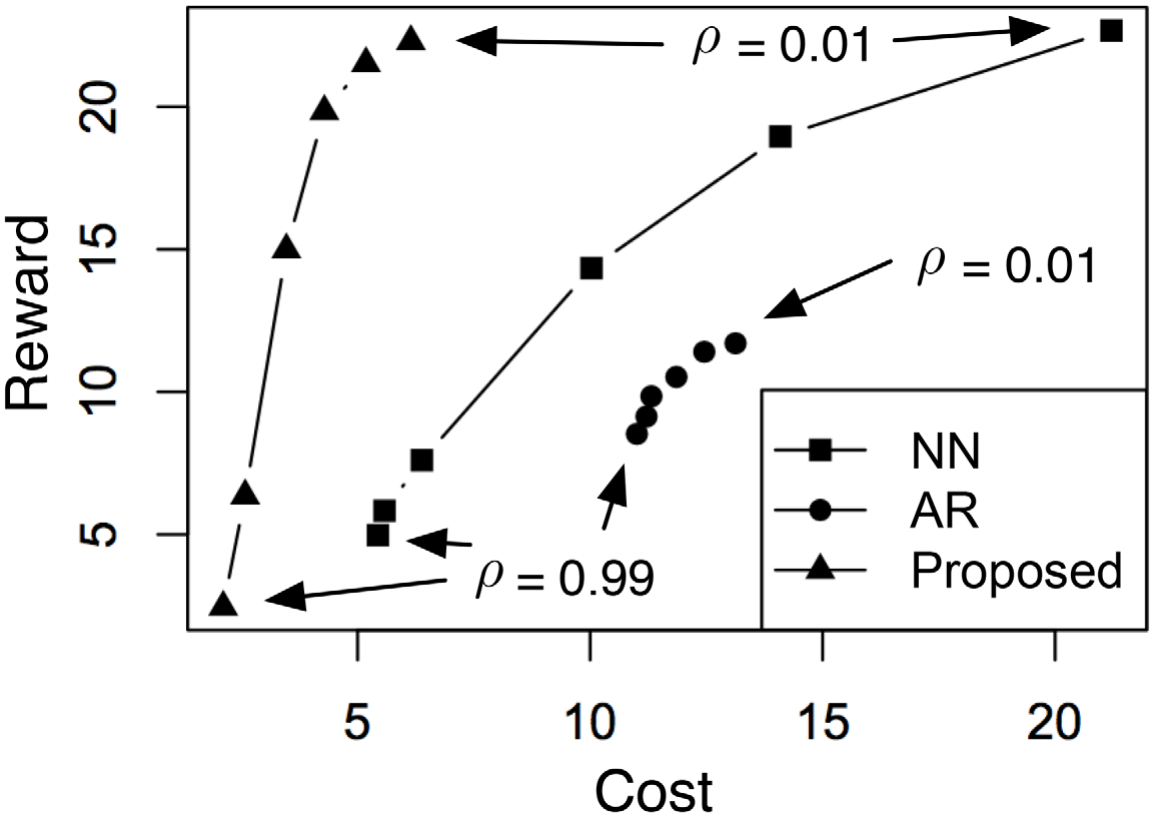
Tradeoff between cost and mismatch using different values of *ρ*. For each method, the point in the upper-right corner corresponds to the case of *ρ* = 0.01, whereas the point in the lower-left corresponds to the case of *ρ* = 0.99.

In Fig 7, we further analyze the tradeoff of using different values of *ρ* where λ is selected using Eq 13. Again, in cases when there’s no λ that satisfies γ(λ) ≥ 0, we select arg max_λ_ γ(λ) instead. The results of average reward versus cost using all three methods are summarized in Fig 7. For each method, the point in the upper-right corner corresponds to the case of *ρ* = 0.01, whereas the point in the lower-left corresponds to the case of *ρ* = 0.99. We note that, in general, the proposed method provides, again, that best overall tradeoff compared to NN and AR, which confirms that the selection of a good generative model is crucial for dynamics engineering. This is also consistent with the observations in Fig 3.

Interestingly, there are also two observations in Fig 7 that are somewhat different from the case of Fig 3. First, while NN seems to be slightly better than AR in Fig 3, it is significantly better in the case of Fig 7. It indicates that, due to the increased difficulty of profit maximization (compared to pattern matching), AR becomes no longer useful. Second, the reward produced by the proposed method is comparable to that of NN, although the proposed method requires much less cost. This suggests that in profit maximization, reducing cost is much easier than increasing reward. This also makes intuitive sense: while it is hard to beat the “Ice Bucket Challenge” in popularity, it might be possible to engineer its marketing campaign such that the cost can be reduced.

#### 4.5.3 Constrained Reward Maximization

To demonstrate the potential benefits of purposeful engineering, we now use a slightly different setting. While for each test sample *i*, we are still given the observation part *X*_*i*_, we enforce an additional constrain that the a solution must produce a cost that is at most *half* of the *actual* cost of sample *i*, i.e., ||*U*_*i*_||_1_, on top of achieving γ ≥ 0, to be considered a *valid* answer. This setting allows us to compare the performance of the profit-maximization algorithms, in terms of reward and cost, against what actually happened in reality.

**Fig 8:**
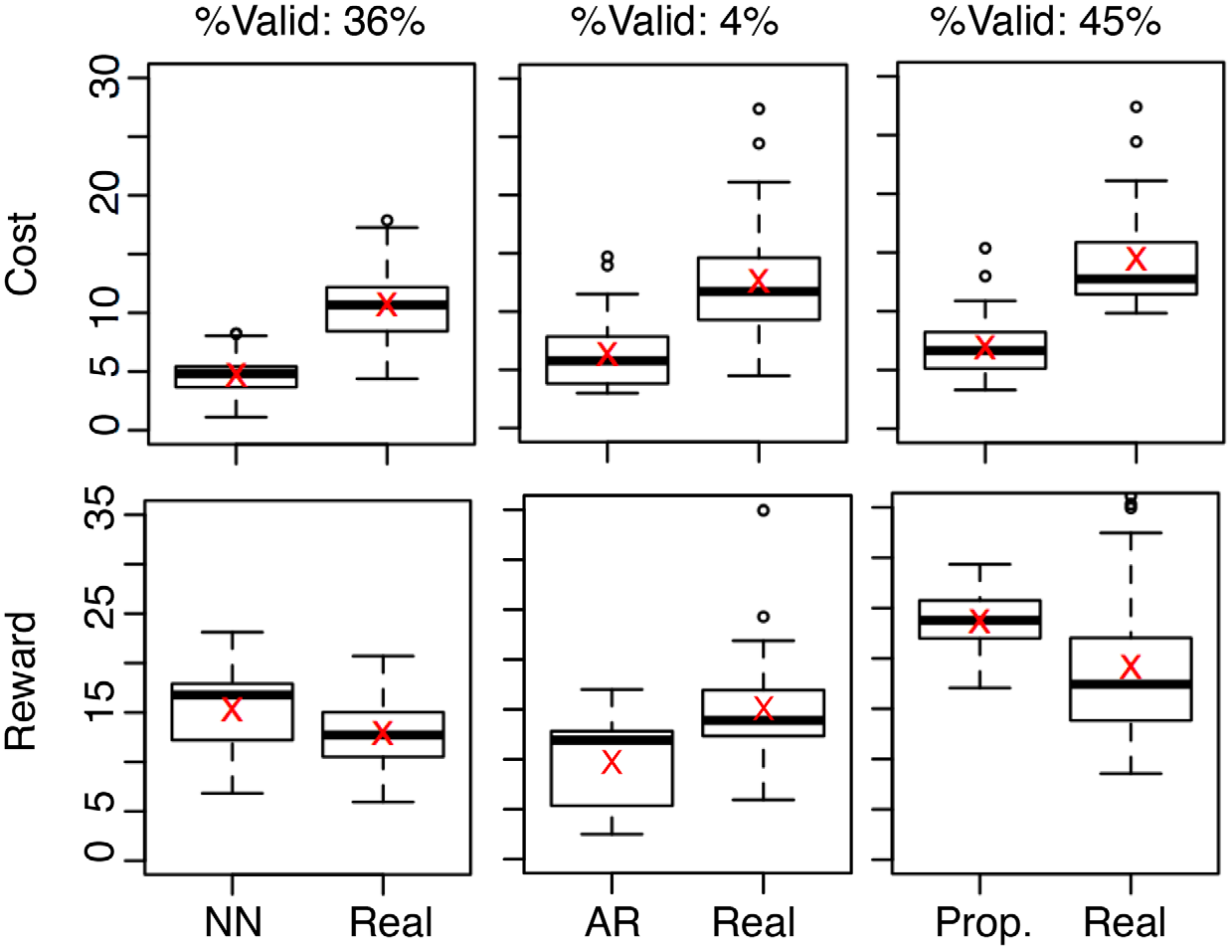
Cost distribution of valid answers using different methods. The cost distribution of each method is contrasted with that of the same set of samples in the real case. The mean values are marked using red crosses.

Fig 8 summarizes the results; again, the fraction of valid answers are annotated at the top and the mean values are marked using red crosses. From the figure, we can make three observations. First, the fractions of valid samples are significantly lower than the case of Fig 4. Indeed, NN produces valid answers for 36% of the test samples, whereas the number is 4% for AR and 45% for the proposed method. This confirms that profit maximization is, in some sense, harder than pattern matching. Second, while all valid solutions from each of the three methods have an average cost lower than half of the corresponding real cases (per our experimental design), these methods result in different reward distributions. Indeed, AR, NN, and the proposed method produce lower, comparable, and higher rewards compared to the real cases, respectively. This confirms that the proposed approach outperforms the two baseline methods and further highlights the profit-maximization potential of the proposed dynamics-engineering method.

#### 4.5.4 Case Study

**Fig 9:**
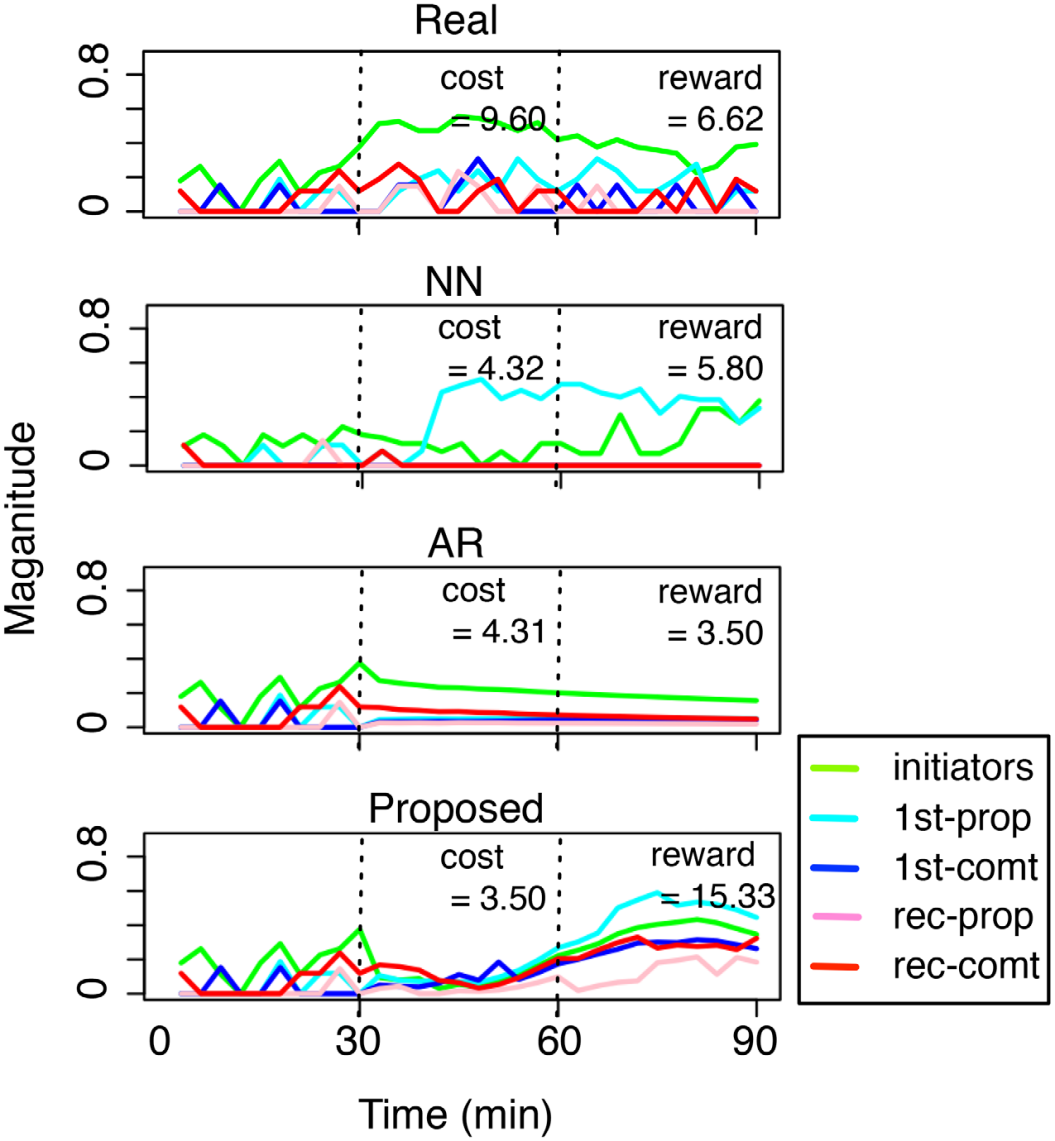
Case study: real versus the suggested dynamics using different methods. A good solution is characterized by low cost (during *t* ∈ [30, 60]) and high reward (during *t* ∈ [60, 90]). The X-axis denotes time (in minutes) and the Y-axis denotes the normalized number of different types of users.

To gain further insights, we pick a test case where all three methods produce valid solutions from the experiment of Fig 8 and plot their suggested solutions in Fig 9. All settings remain the same as the case of Fig 5. To compare among different solutions (i.e., NN, AR, and Proposed), we note that the ideal profit maximization should achieve both low cost during *t* ∈ [30, 60] and high reward during *t* ∈ [60, 90].

From Fig 8, we can see that, although the solutions from all three methods (NN, AR, and the proposed method) have costs lower than half of the real case, their rewards are quite different. For NN, the reward of its solution is comparable to that of the real case. Given that it also has a lower cost compared to the real case, this solution is not too bad. For AR, while the reward is even lower, the real issue is that the solution dynamics doesn’t look real: in fact, its solution validity γ ≈ 0.004, i.e., barely passes 0. Finally, the proposed method not only produces a recommendation that has a low cost, but also a reward higher than the real case. A closer inspection shows that although the magnitude of the intervention dynamics (i.e., the second part) is generally low, it seems to contain interesting interactions because the recommendation includes different key roles at different stages: recurring commentators (red) around time *t* = 35, first-time propagators (dark blue) around *t* = 50, and then the dominating first-time propagators after *t* ≥ 60. All these interactions reflect the patterns (i.e., the filters *W*’s in Eqs 9 and 8) of different temporal scales that are learnt directly from data. This is why the proposed method is able to recommend solutions with low cost and high reward, while still making the suggested dynamics follow the intrinsic temporal dependencies from the data.

## 5 Discussion

### 5.1 Pattern Matching vs. Profit Maximization

The merits of the proposed pattern matching and profit maximization are quite different. Indeed, from Fig 4, the proposed pattern matching is capable of producing valid solutions for 98% of the test samples, while reducing the cost by an average of 82% with a minor mismatch within 5%. On the other hand, from Fig 8, the proposed profit minimization is capable of producing valid solutions for 45% of the test samples while improving the reward by an average of 27% with less than half of the original cost. Such a difference originates from the two tasks’ different goals and formulations: pattern matching (Eq 3) aims at matching a given pattern with the lowest cost, whereas profit maximization aggressively maximizes reward and minimizes cost.

Further, such a difference in formulation implies a difference in the fundamental *difficulties* of two tasks. More importantly, profit maximization is significantly more difficult because while the “cost” has a natural lower bound (i.e., zero), the “reward”, in principle, does not have any upper bound. In other words, unless the parameter λ is assigned perfectly, it is very easy to either obtain an invalid solution or a low-score solution. Therefore, in many engineering cases (e.g., marketing promotion), although profit maximization may be more desirable, in practice, pattern matching may be more useful.

The above analysis is confirmed by our experimental results in two ways. First, by comparing Fig 2 with Fig 6, we see that it is significantly harder to generate a valid solution in profit maximization. Indeed, compared to the case of pattern matching (Fig 2), the area above the horizontal line γ ≥ 0 is much smaller in the case of profit maximization (Fig 6). Also, compared to the case of pattern matching, the range of λ that corresponds to γ ≥ 0 (λ ≈ 0.01) is much more narrow than the case of Fig 2 (λ ∈ [0.01, 1]). It suggests that it is harder to select a good value for the parameter λ in the case of profit maximization. Second, by comparing Fig 3 with Fig 7, we see that while pattern matching is capable of reducing both cost and mismatch, profit maximization is more capable of achieving a reasonable reward using low cost, compared to achieving a very high reward using moderate cost. Indeed, from Fig 7, we see that although the cost of the proposed solution is much lower than the case of the NN (i.e., nearest-neighbor) baseline, their highest possible rewards are only comparable.

These differences among the two tasks have practical implications on their real applications. First, if the ideal outcome pattern is given, pattern matching is the better option because according to Fig 4, there is a 98% chance that a valid solution will be produced with low cost and mismatch. Second, if the ideal pattern is not given, then according to Fig 8, there is a 45% chance that a valid solution will be produced. In this case, a moderately high reward with a low cost can be expected.

### 5.2 Future Directions

We believe this work is only the first step toward a new field with great potential, namely, *data-driven dynamics engineering*. While significant follow-up work can be built on the foundation this work offers, we would like to point out three directions that seem to hold the most promise. The first direction involves exploring different combinations of generative models and score functions as mentioned in Eq 5. Although we derive our solution based on a particular model (RCBM) and evaluate it using two specific score functions (Eqs 3 and 4), other combinations can introduce equally, if not more, important engineering applications.

The second direction involves building a complete tool chain of dynamics engineering. In that sense, this work only accomplishes the very first component, i.e., figuring out what the ideal intervention should be. Two other important components in the tool chain are (1) how to *implement* that intervention most effectively and most efficiently and (2) how to efficiently *validate* the effectiveness of intervention given limited resources (i.e., using field experiments).

We note that this work serves as the foundation for the other two components by providing a principled, data-driven method to offer an ideal intervention and its anticipated outcome. Using such information, the engineer gets to eliminate the need for trial-and-error among all possible interventions. Instead, he or she can focus on the implementation and validation perspectives of dynamics engineering, both of which justify an in-depth investigation on their own right. If we look back at the trajectory of how the tool chain of the Integrated-Circuit industry (worth $300B as of 2016) was built, we are right at the very beginning of it.

Finally, although we look exclusively at social dynamics, the formulation proposed in this work applies generally to any multi-dimensional time series. Indeed, applications such as mobile context-aware computing, computational economics, and healthcare could potentially also use this framework to engineer their own dynamics problems. In this sense, we believe that developing a discipline for data-driven dynamics engineering is full of potential.

## 6 Conclusion

In this paper, we have defined a new problem that is full of long-term potential, i.e., the data-driven engineering of social dynamics. To the best of our knowledge, this work brings the following new contributions:

1. *Data-Driven Dynamics Engineering*: We propose a framework for data-driven dynamics engineering. Our formulation is precise yet general and includes pattern matching and profit maximization as special cases. Using a deep-learning model, we derive a solution based on convex relaxation and quadratic-programming transformation, which is efficient with guaranteed global convergence. We also propose a data-driven evaluation method instead of the time-consuming and expensive field experiments [17].
2. *Pattern Matching vs. Profit Maximization*: Using a Twitter dataset, the proposed pattern matching generates valid solutions in 98% of the test cases, achieving an average cost reduction of 82% with a mismatch within 5%. Moreover, the proposed profit maximization generates valid solutions in 44% of the cases, achieving an average reward improvement of 27% with a cost reduction of at least 50%. We further report and analyze the interesting tradeoffs in how to chose among these two tasks, as well as the factors that are critical to real-world dynamics engineering applications, including solution validity, pattern mismatch, intervention cost, and outcome popularity.

Since the proposed formulation applies generally to any multi-dimensional time series, we believe this approach can be also applied to the engineering tasks of other applications. We hope this work serve as the first step in this new field of great potential.

## Supporting Information

S1 Appendix Proof of Theorem 1 and 2; derivation of the baseline solution based on the autoregressive (AR) model. (PDF)
