# Supplementary material for: Data-Driven Engineering of Social Dynamics: Pattern Matching and Profit Maximization
Source: PLoS One. 2016 Jan 15;11(1):e0146490. doi: 10.1371/journal.pone.0146490 (PMC4714878; doi:10.1371/journal.pone.0146490)
Supplement: S1 Appendix — (PDF) [file pone.0146490.s001.pdf]

APPENDIX

A. QP TRANSFORMATION

Since the objective function of Equation 11 is quadratic with only linear constraints, in principle, there exists a QP problem that is equivalent to Equation 11. The explicit form of this QP transformation is given by the following theorem.

Theorem 1. *Let* $z = [\mathbf{y}^T\ \mathbf{h}_1^T\ \mathbf{h}_2^T\ \mathbf{s}^T]^T$*, then the quadratic programming problem*

$$\begin{array}{ll} \arg\min_z & \frac{1}{2}z^TQz + p^Tz \\ \text{s.t.} & Az \geq 0, \end{array}$$

is equivalent to the problem in Equation 11, where

$$\begin{aligned}
Q &= \begin{bmatrix} I_{m_y} - 2\lambda B & -\hat{I}_Y G_1 & \mathbf{0} & \mathbf{0} \\ -(\hat{I}_Y G_1)^T & G_1^T G_1 & \mathbf{0} & \mathbf{0} \\ \mathbf{0} & \mathbf{0} & G_2^T G_2 & -G_2 \\ \mathbf{0} & \mathbf{0} & -G_2^T & I_{m_s} \end{bmatrix} \\
A &= \begin{bmatrix} I_{m_y} & \mathbf{0} & \mathbf{0} & \mathbf{0} \\ \mathbf{0} & I_{m_{h_1}} & \mathbf{0} & \mathbf{0} \\ \mathbf{0} & \mathbf{0} & I_{m_{h_2}} & \mathbf{0} \\ \mathbf{0} & -I_{m_{h_1}} & \mathbf{0} & M \\ \mathbf{0} & M^T & \mathbf{0} & -I_{m_s} \end{bmatrix} \\
p &= [-\lambda d^T, \tfrac{\sigma_1^2}{\beta_1}\mathbf{1}_{1\times m_{h_1}} - ((\hat{I}_X G_1)^T\mathbf{x})^T, \tfrac{\sigma_2^2}{\beta_2}\mathbf{1}_{1\times m_{h_2}}, \mathbf{0}_{1\times m_s}].
\end{aligned}$$

As mentioned before, bold-faced letters (e.g., $\mathbf{y}$ and $\mathbf{s}$) denote the vectorization of matrices with the same capital letters (e.g., Y and S), while $\mathbf{h}_i$ denotes the vector concatenation of $\{h_{ik}\}_{k=1}^{K_i}$ for the i-th level. In the above theorem, we define $\hat{I}_X = I_D \odot [I_{T_x}\ \mathbf{0}_{T_x\times T_y}]$ and $\hat{I}_Y = I_D \odot [\mathbf{0}_{T_y\times T_x}\ I_{T_y}]$, where $\odot$ is the Kronecker product. Also, G_i denotes the matrix representation of the convolution operations $\sum_k W_{ik} \otimes h_{ik}$, which is defined as:

$$G_i = \begin{bmatrix} G_{11}^{(i)} & G_{12}^{(i)} & \dots & G_{1K_1}^{(i)} \\ \vdots & \vdots & \vdots & \vdots \\ G_{D1}^{(i)} & G_{D2}^{(i)} & \dots & G_{DK_1}^{(i)} \end{bmatrix}$$

where $G_{lk}^{(i)}$ is the Toeplitz matrix of a l-th column of the filter matrix W_{ik} [22]. Finally, $M \in R^{m_{h1}\times m_s}$ is defined as $M_{i,j} = 1$ if $j = \lceil i/c \rceil$ and $M_{i,j} = 0$ otherwise. The proof of this theorem is provided in Appendix B. We note that this proposed quadratic programming problem is convex, it is guaranteed to find an approximate solution in polynomial time. In our experiments, the vector z consists of around 1000 variables and even so the problem gets solved in just a few seconds.

B. PROOF OF THEOREM 1

LEMMA 1. *Given* $X \in R^{D\times T_x}$, $Y \in R^{D\times T_y}$*, define*

$$\tilde{I}_X = [I_{T_x\times T_x}\ \mathbf{0}_{T_x\times T_y}],\ \tilde{I}_Y = [\mathbf{0}_{T_y\times T_x}\ I_{T_y\times T_y}],$$

and

$$\hat{I}_X = I_D \odot \tilde{I}_X,\ \hat{I}_Y = I_D \odot \tilde{I}_Y,$$

where $\odot$ *is the Kronecker product [22], then we have*

$$\hat{I}_X\mathrm{vec}([X\ Y]) = \mathrm{vec}(X),\ \text{and}\ \hat{I}_Y\mathrm{vec}([X\ Y]) = \mathrm{vec}(Y),$$

and

$$\hat{I}_X^T\hat{I}_X + \hat{I}_Y^T\hat{I}_Y = I_{D\cdot(T_x+T_y)}.$$

PROOF OF LEMMA 1. Using property of the Kronecker product that $B^T \odot A\mathrm{vec}(X) = \mathrm{vec}(AX^TB^T)$ [22], we have

$$\begin{aligned}
\hat{I}_X\mathrm{vec}([X\ Y]) &= I_D \odot \tilde{I}_X\mathrm{vec}([X\ Y]) \\
&= \mathrm{vec}(\tilde{I}_X[X\ Y]^T I_D) \\
&= \mathrm{vec}([I_{T_x\times T_x}\ 0_{T_x\times T_y}][X\ Y]^T) \\
&= \mathrm{vec}(X).
\end{aligned}$$

For the second claim, we compute

$$\begin{aligned}
\hat{I}_X^T\hat{I}_X &= (I_D \odot \tilde{I}_X)^T(I_D \odot \tilde{I}_X) \\
&= (I_D \odot \tilde{I}_X^T)(I_D \odot \tilde{I}_X) \\
&= I_D \odot (\tilde{I}_X^T\tilde{I}_X).
\end{aligned}$$

Similarly $\hat{I}_Y^T\hat{I}_Y = I_D \odot \tilde{I}_Y^T\tilde{I}_Y$ and thus

$$\hat{I}_X^T\hat{I}_X + \hat{I}_Y^T\hat{I}_Y = I_D \odot (\tilde{I}_X^T\tilde{I}_X + \tilde{I}_Y^T\tilde{I}_Y) = I_D \odot I_{(T_x+T_y)} = I_{D\cdot(T_x+T_y)}.$$

□

LEMMA 2. *Given filter matrices* W_{ik}*, then the vectorization of its convolution operation with respect to* h_{ik} *can be expressed as*

$$\mathrm{vec}(\sum_{k=1}^{K_1} h_{ik}\otimes W_{ik}) = \begin{bmatrix} G_{11}^{(i)} & G_{12}^{(i)} & \dots & G_{1K_1}^{(i)} \\ \vdots & \vdots & \vdots & \vdots \\ G_{D1}^{(i)} & G_{D2}^{(i)} & \dots & G_{DK_1}^{(i)} \end{bmatrix} \begin{bmatrix} h_{i1}^T \\ h_{i2}^T \\ \vdots \\ h_{iK_1}^T, \end{bmatrix}$$

where $G_{lk}^{(i)}$ *is the Toeplitz matrix representing the operation* $\otimes$ *w.r.t a single filter* $w_{lk}^{(i)}$ *in* l*-th row of the filter matrix* W_{ik} *[22], i.e.,* $G_{lk}^{(i)}h_{ik}^T = (w_{lk}^{(i)} \otimes h_{ik})^T$.

PROOF OF LEMMA 2. Given that $w_{lk}^{(i)}$ is the l-th row of the filter matrix W_{ik}, we have

$$\begin{aligned}
\mathrm{vec}(\textstyle\sum_{k=1}^{K_1} h_{ik}\otimes W_{ik}) &= \textstyle\sum_{k=1}^{K_1} \begin{bmatrix} G_{1k}^{(i)}h_{ik}^T \\ G_{2k}^{(i)}h_{ik}^T \\ \vdots \\ G_{Dk}^{(i)}h_{ik}^T \end{bmatrix} \\
&= \begin{bmatrix} G_{11}^{(i)} & G_{12}^{(i)} & \dots & G_{1K_1}^{(i)} \\ \vdots & \vdots & \vdots & \vdots \\ G_{D1}^{(i)} & G_{D2}^{(i)} & \dots & G_{DK_1}^{(i)} \end{bmatrix} \begin{bmatrix} h_{i1}^T \\ h_{i2}^T \\ \vdots \\ h_{iK_1}^T \end{bmatrix}
\end{aligned}$$

□

PROOF OF THEOREM 1. Using Lemma 1 and 2, we can compute

$$\begin{aligned}
&\|[X\ Y] - \textstyle\sum_{k=1}^{K_1} h_k \otimes W_{1k}\|_F^2 = \|\mathrm{vec}([X\ Y]) - G_1h_1\|_F^2 \\
&= \|\mathrm{vec}(X) - \hat{I}_X G_1 h_1\|_F^2 + \|\mathrm{vec}(Y) - \hat{I}_Y G_1 h_1\|_F^2 \\
&= \mathbf{h}_1^T G_1^T G_1 \mathbf{h}_1 + \mathbf{y}^T\mathbf{y} - 2\mathbf{y}^T\hat{I}_Y G_1\mathbf{h}_1 - 2\mathbf{x}^T\hat{I}_X G_1\mathbf{h}_1 + \mathbf{x}^T\mathbf{x}.
\end{aligned}$$

Similarly,

$$\begin{aligned}
&\|S - \textstyle\sum_{k=1}^{K_2} h_{2k} \otimes W_{2k}\|_F^2 = \|\mathrm{vec}(S) - G_2h_2\|_F^2 \\
&= \mathbf{h}_2^T G_2^T G_2\mathbf{h}_2 + \mathbf{s}^T\mathbf{s} - 2\mathbf{s}^T G_2\mathbf{h}_2.
\end{aligned}$$

Expand these two terms in the objective function of equation 11 and sum up everything, we have the desired form in Theorem 1. □

C. AR BASELINE

The order-p ARMA model is defined by $x_t = \sum_{i=1}^{p} \Phi_i x_{t-i} + \epsilon_t$,, where $x_i \in R^{D\times 1}$ are multivariate features and $\epsilon_t \sim \mathcal{N}(0,\ \Sigma)$ is the i.i.d. Gaussian noise. We shall derive and solve the equation 8 based on order-p ARMA model. First we derive the log likelihood of order-p ARMA.

LEMMA 3. *Let $\tilde{\Phi}_i = \Sigma^{-1/2}\Phi_i$, and define the Toeplitz matrix of $[-\tilde{\Phi}_p,\ -\tilde{\Phi}_{p-1},\ \ldots, -\tilde{\Phi}_1,\ I_D]$ by shifting $\tilde{\Phi}$ $T_x - p$ times, i.e.,*

$$\hat{\Phi} = \begin{bmatrix} -\tilde{\Phi}_p & \ldots & -\tilde{\Phi}_1 & \Sigma^{-1/2} & & & \\ & -\tilde{\Phi}_p & \ldots & -\tilde{\Phi}_1 & \Sigma^{-1/2} & & \\ & & \ddots & & \ddots & \ddots & \\ & & & -\tilde{\Phi}_p & \ldots & -\tilde{\Phi}_1 & \Sigma^{-1/2} \end{bmatrix}.$$

Then

$$\sum_{t=p+1}^{T_x} \log P(x_t|x_{t-i}\ i = 1,\ \ldots,\ p) = -\|\hat{\Phi}[x_1^T,\ \ldots,\ x_{T_x}^T]^T\|_F^2/2.$$

THEOREM 2. *Assume that the order-p AR time series is divided in to three sections 1) the given observation $x = [x_1^T\ x_2^T \ldots x_p]^T$ 2) the intervention $u = [x_{p+1}^T\ \ldots x_{p+T_u}^T]^T$ and the outcome $v = [x_{p+T_u+1}^T\ \ldots x_{p+T_u+T_v}^T]^T$ with a reference pattern v_{ref}. Let $Q = \hat{\Phi}^T\hat{\Phi} = \begin{bmatrix} Q_{11} & Q_{12} \\ Q_{21} & Q_{22} \end{bmatrix}$ where $Q_{11} \in R^{T_x\times T_x}$, $Q_{22} \in R^{T_y\times T_y}$ and $y^T = [u^T v^T]$. Then the problem*

$$\begin{array}{ll} \text{argmax}_y & \log P(y|x) - \lambda(\|v - v_{ref}\|_F^2 + \rho\|u\|_1) \\ \text{s.t.} & y \geq 0 \end{array}$$

is equivalent to a quadratic programming problem

$$\begin{array}{ll} \text{argmin}_y & \frac{1}{2}y^T(Q_{22} + 2\lambda \begin{bmatrix} 0 & 0 \\ 0 & I_v \end{bmatrix})y + (Q_{21}x - \lambda d)^T y \\ \text{s.t.} & y \geq 0 \end{array}$$

where $d^T = [-\rho\mathbf{1}^T,\ 2v_{ref}^T]$.

PROOF. Both the lemma and theorem are proved by direct calculation. □
